# Immune response in cutaneous leishmaniasis patients with healing *vs*. non-healing lesions

**Published:** 2020-06

**Authors:** Akram Miramin-Mohammadi, Amir Javadi, Seyed Ebrahim Eskandari, Hossein Mortazavi, Mahmoud Nateghi Rostami, Ali Khamesipour

**Affiliations:** 1 Center for Research and Training in Skin Diseases and Leprosy, Tehran University of Medical Sciences, Tehran, Iran; 2 Department of Social Medicines, Qazvin University of Medical Sciences, Qazvin, Iran; 3 Department of Dermatology, Tehran University of Medical Sciences, Razi Hospital, Tehran, Iran; 4 Department of Parasitology, Pasteur Institute of Iran, Tehran, Iran

**Keywords:** Cutaneous leishmaniasis, Soluble *Leishmania* antigen (SLA), Immune response, Interferon gamma (IFNγ), Interleukin 10 (IL-10), Interleukin 5 (IL-5)

## Abstract

**Background and Objectives:**

The outcome of *Leishmania* infection mainly depends upon the *Leishmania* species which causes the disease and the generation of the type of host immune response, the healing process and protection in leishmaniasis depends upon induction of Th1 response. In this study, the Th1/Th2 cytokine profile in cutaneous leishmaniasis (CL) is evaluated.

**Materials and Methods:**

This study was carried out in leishmaniasis clinic of CRTSDL, TUMS, during March 2018 to March 2019. Peripheral blood mononuclear cells (PBMC) of volunteers with active healing and non-healing lesion (s) of cutaneous leishmaniasis (CL), volunteers with and without history of CL were cultured and stimulated with Soluble *Leishmania* antigen (SLA). The supernatants were collected and the levels of IFN-γ, IL-5 and IL-10 were titrated using ELISA method.

**Results:**

The results showed a significantly higher levels of IFN-γ in volunteers with active CL healing form (*p*<0.005), history of CL (*p*<0.005) than healthy volunteers. A significantly (*p*<0.005) higher level of IFN-γ was seen in volunteers with active healing form of lesion than non-healing form. There was a significantly (*p*<0.005) higher level of IL-10 in volunteers with a history of non-healing form and active non-healing form of CL. There was no significant difference in IL-5 production in PBMC of different groups.

**Conclusion:**

IFN-γ production starts at early stage of cutaneous leishmaniasis and enhance during course of lesion healing, IFN-γ level is significantly higher in all patients compared to healthy volunteers, IFN-γ is significantly higher in patients with healing form than non-healing form of lesion.

## INTRODUCTION

Leishmaniasis is endemic in 102 countries and is the main health problem in some of the endemic regions which are the poorest areas of the world. Leishmaniasis is endemic in 14 of 22 WHO/EMRO region countries. Annually, 200,000–400,000 people develop visceral leishmaniasis, and 700,000–1,200,000 with cutaneous leishmaniasis (CL). Recently the disease spread to some non-endemic areas. The burden of the disease (DAILYs) is reported to be 3.3 million, clinical manifestations include CL, mucocutaneous (MCL), visceral (VL), and post-kala-azar dermal leishmaniasis (PKDL) (1–3). The host immune response and the parasite species determine the outcome of *Leishmania* infection. CL is a self-healing lesion, healing process takes place in less than a year if the causative agent is *L. major* and about 2 years if the causative agent is *L. tropica* (4).

In mouse model of *L. major* infection, the type of T-cell response determines the outcome of the infection; in resistant mice, a Th1 response is induced with production of IFN-γ, the lesion cure and the animals are protected against challenge, which is somehow similar to human CL, while in susceptible BALB/c mice, Th2 response is generated and a high level of IL-4 is produced, IFN-γ production is down regulated, and every infected mouse is succumbed to the disease. Although, the generation of Th1 type of response is related to cure and protection and induction of Th2 type of response is accompanied with progress of the disease and death in murine model but susceptibility and resistant in human leishmaniasis is not yet well defined (10-5). Generally, presence of cells that produce IFN-γ occurs in healing form of CL, whereas in non-healing form of CL and mucosal lesions there is a mixture of Th1/Th2 cytokines with an abundance of IL-4 and IL-10 (11–15).

In this study, PBMC were collected from patients with active lesion (s), healing and non-healing forms of CL; healing form of lesion refers to the patient whose lesion heals with or without treatment, non-healing form of lesion refers to the lesion which does not respond to at least two full courses of systemic injections of antimonite derivatives, also PBMC were collected from volunteers with history of CL, and healthy volunteers with no history of leishmaniasis. The collected PBMC were stimulated with SLA and the levels of IFN-γ, IL-10 and IL-5 production were compared.

## MATERIALS AND METHODS

### Ethical consideration and study groups.

This study was approved by the Ethical Committee of Tehran University of Medical Sciences (TUMS) and completed at the Center for Research and Training in Skin Diseases and Leprosy (CRTSDL), during March 2018 to March 2019. The potential candidates were interviewed and informed about the objectives and the procedure of the study and the one who was willing to participate, donate blood sample and sign an informed consent was recruited. Management of CL lesion including diagnosis, treatment of the patients were done free of charge.

The following volunteers were recruited, the first group consisted of 10 healthy volunteers with no history of leishmaniasis, leishmanization or vaccination against leishmaniasis, (control group), the second group consisted of 10 volunteers with non-healing active CL lesions (the onset of the lesion more than 2 years with history of at least 2 courses of Glucantime treatment), the third group consisted of 10 volunteers with healing form of active CL lesion (s), the onset of lesion in this group was less than one year, the fourth group consisted of 10 volunteers with history of non-healing CL lesion (completely cured), and the last group consisted of 10 volunteers with history of healing form of CL (completely cured). The volunteers were healthy other than CL according to physical examination by a physician, male/female, age 12–70 years old.

Diagnosis was based on observation of amastigote form of *Leishmania* using Giemsa stained smear and/or growth of promastigotes in NNN culture (16). Identification of *Leishmania* causative agent was done using PCR method (16, 17).

### PCR method.

PCR was carried out using the primers for *Leishmania*-specific pair fragment of kinetoplast DNA. The sequences of the two synthetic oligonucleotide primers of KDNA pattern are F: (5′ TCGCAGAACGCCCCTACC 3′) and R: (5′ AGGGGTTGGTGTAAAATAGG 3′). Amplification was performed in a thermal cycler (ASTEC-PC818) 38 cycles of denaturation at 94°C for one minute, annealing at 60°C for 45 second, extension at 72°C for one minute and final extension at 72°C for 7 minutes. Two standard samples of parasites of *L. major* and *L. tropica* and a negative control sample were used to monitor the reaction. PCR products were run in 1.5% agarose gel electrophoresis and observed by UVdoc system (Doc-008.XD). DNA replication pattern of *Leishmania* produced bands for *L. major*/600 bp and for *L. tropica*/800 bp (17).

### Soluble *Leishmania* antigen (SLA) preparation.

*L. major* (MRHO/IR/75/ER), which was used for leishmanization and preparation of experimental *Leishmania* vaccine and leishmanin was used in this study. Parasites were grown in NNN medium and sub passaged in RPMI 1640 media supplemented with 10% FCS and penicillin/streptomycin (Complete RPMI). Promastigotes were harvested at stationary phase, washed 3 times and adjusted to 1.2 × 10^9^ parasite/ml in buffer solution. Soluble *Leishmania* Antigen (SLA) was prepared using the protocol developed by Scott et al. (18) with minor modifications, briefly, the parasites were harvested at stationary phase and washed 4 times using HEPES-sucrose buffer (10 mM, 10% w/v, pH = 7.4). Then, the number of promastigotes was adjusted to 1.2 × 10^9^/ml in buffer solution containing enzyme inhibitor cocktail, 50 μl/ml (Sigma, St. Louis, USA). The parasites were lysed using freeze-thaw method followed by probe sonication in an ice bath. The supernatant of the centrifuged lysate parasites was collected, dialyzed against buffer solution, sterilized using a 0.22 μm membrane and stored at −70°C until use. The protein concentration of the preparation was determined using BCA protein assay method (Thermo Scientific, USA).

### Blood sampling collection.

Heparinized blood samples (10 mL) were collected from every volunteer. Blood samples were diluted 1:1 with RPMI. The diluted blood sample was overlaid gradually with Ficoll-hypaque (30–40% of blood volume) in 50 ml disposable centrifuge tubes. The peripheral mononuclear cells (PBMCs) were then separated by centrifugation gradient at 800 g for 30 minutes at room temperature. After centrifugation, the interface containing mononuclear cells was collected with a Pasteur-pipette and transferred into a 15 ml disposable conical centrifuge tube. Cells were washed two times with RPMI + 3% FCS using 490 g centrifugation for 10 minutes at 5ºC and resuspended in Complete RPMI (10, 11).

### Cell culture of PBMC.

Fresh PBMCs were cultured in CRPMI in U-bottom 96 well culture plates (Nunc, Denmark). Each well was contained 2 × 10^5^ cells in 200 μl volume per well in triplicate. The cells were stimulated with either Phytohaemagglutinin (PHA) (5 μg/ml) or SLA (10 μg/ml), or no stimulation only with culture media alone as a negative control. Then, the cells were incubated for 72 h at 37°C in 5% CO_2_. After 72 hours incubation, 150 μl of the supernatants were carefully collected from each well and the triplicates were pooled and kept at −80°C until used (10, 11).

### Cytokine measurements.

Cytokines (IL-5, IL-10 and IFN-γ) in supernatants were measured using an enzyme-linked immunosorbent assay and the biotin-avidin system according to the manufacturer’s guidelines (Bioscience Kit). Results are expressed in pg/ml (median ± IQR) of triplicates.

### Statistical analysis.

The normality of numerical variables was assessed with Shapiro-Wilk test. Data was expressed as the mean ± SD or median ± IQR, and percentage. Chi-square test and Fisher’s exact test was used to examine the relation between qualitative variables. The paired t-test/Wilcoxon signed-rank test was used to measure changes inter-groups. One-way ANOVA with followed post-hoc test (Bonferroni) was used to multiple comparisons between groups. P value of <0.05 was considered as significant. The SPSS Version 16 (SPSS Inc., Chicago, IL, USA) software was used for all statistical analyses.

## RESULTS

Demographic and lesions characteristics of the recruited volunteers are presented in Table 1.

**Table 1.** Demographic and lesion characteristics of volunteers.

	**Healthy**	**Active lesion (non-healing form)**	**Active lesion (healing form)**	**History of CL (non-healing form)**	**History of CL (healing form)**
Number of volunteers	10	10	10	10	10
Mean of Age (Year)	45	44.3	35.6	25.3	33.2
Gender (M/F)	8/2	4/6	2/8	2/8	5/5
Number of lesion	-	14	38	12	22
Location of lesion					
Upper limb	-	4	2	6	6
Lower limb	-	-	8	-	2
Trunk	-	-	2	-	-
Face	-	4	-	3	3
Duration of thelesion	-	22.1	2.3	17	4.3
(mean in months)					
Species L.m/L.t	-	6/4	8/2	5/5	7/5

### Level of IFN-γ in volunteers’ PBMC stimulated with SLA.

**Fig. 1.**
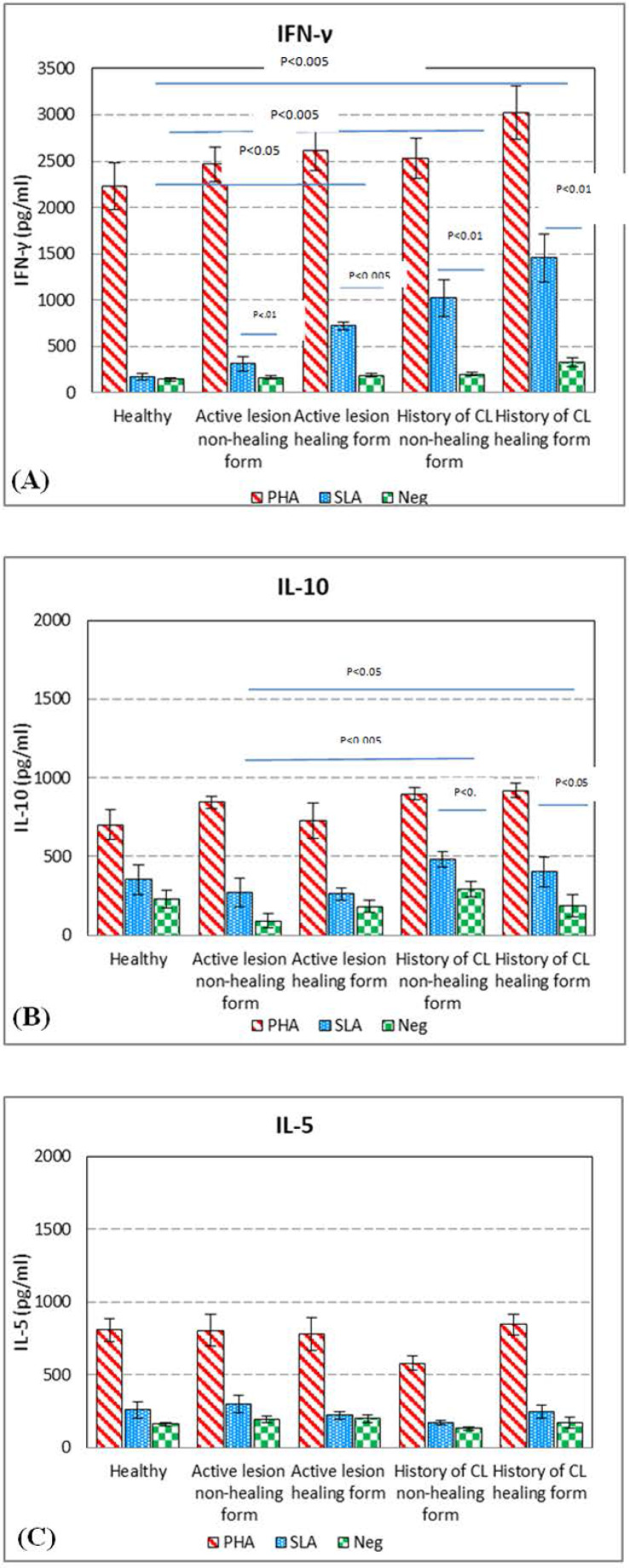
Cytokine levels in culture supernatants of PBMC of volunteers with active lesion (non-healing form) and active lesion (healing form), history of CL (non–healing form), history of CL (healing form), and healthy volunteers stimulated with either PHA, SLA (soluble *Leishmania* antigen), or no stimulation as control. (A) IFN-γ level in culture (B) IL-10 level in culture (C) IL-5 level in culture

The volunteers’ PBMC were collected and stimulated with SLA and the supernatants were used to titrate the level of IFN-γ, the level of IFN-γ in healthy volunteers with SLA stimulation and without stimulation was 172.3.7 ± 96.4 and 142.3 ± 46.3, respectively, the difference was not significant. The level of IFN-γ in volunteers with active lesion (non-healing form) with SLA stimulation and without stimulation was 310.1 ± 241.3 and 160.6 ± 59.1, respectively, the difference was significant (*p*<0.01). The level of IFN-γ in volunteers with active lesion (healing form) with SLA stimulation and without stimulation was 719.5 ± 137.0 and 188.1 ± 46.3, respectively, the different was significant (*p*<0.005). The level of IFN-γ in supernatants of PBMC collected from volunteers with history of CL (non-healing form) with SLA stimulation and without stimulation was 1,021.3 ± 613.3 and 201.3 ± 70.0, respectively, the difference was significant (*p*<0.01). The level of IFN-γ in supernatants of PBMC collected from volunteers with history of CL (healing form) with SLA stimulation and without stimulation was 1,459.0 ± 818.8 and 331.7 ± 154.4, respectively, the different was significant (*p*<0.01). There was no significant difference between the level of IFN-γ of healthy volunteers and active lesion (non-healing form) after stimulation. There was a significant (P<0.005) difference between the level of IFN-γ in active lesion (healing form) and healthy volunteers after stimulation. There was a significant (P<0.005) difference between the level of IFN-γ in volunteers with history of CL (non-healing form) and healthy volunteers after stimulation. There was a significant difference (P<0.005) between the level of IFN-γ in supernatants of PBMC of healthy volunteers compared with volunteers with history of CL (healing form) after stimulation. There was a significant (P<0.005) difference between the level of IFN-γ in volunteers with active lesion (healing form) and active lesion (non-healing form) after stimulation. There was a significant (P<0.01) difference between the level of IFN-γ in volunteers with active lesion (non-healing form) and volunteers with history of CL (non-healing form) after stimulation. There was a significant (P<0.05) difference between the level of IFN-γ in volunteers with active lesion (healing form) and volunteers with history of CL (healing form) after stimulation. There was a significant (P<0.005) difference between the level of IFN-γ volunteers with history of CL (healing form) and volunteers with active lesion (non-healing form) after stimulation but there was no significant difference between the level of IFN-γ in volunteers with history of CL (non-healing form) and volunteers with history of CL (healing form) after stimulation (Fig. 1A).

### Level of IL-10 in volunteers’ PBMC, stimulated with SLA.

The volunteers’ PBMC were collected and stimulated with SLA and the supernatants were used to titrate the level of IL-10. The level of IL-10 in healthy volunteers with SLA stimulation and without stimulation was 351.9 ± 291.8 and 228.8 ± 175.2, respectively, the different was not significant. The level of IL-10 in active lesion (non-healing form) with SLA stimulation and without stimulation was 269.0 ± 241.2 and 91.1 ± 99.8 respectively, the different was no significant. The level of IL-10 in volunteers with history of CL (non-healing form) with SLA stimulation and without stimulation was 479.4 ± 158.2 and 289.5 ± 155.3, respectively, the difference was significant (P<0.05). The level of IL-10 in volunteers with history of CL (healing form) with SLA stimulation and without stimulation was 419.9 ± 303.2 and 184.8 ± 221.4, respectively, the different was significant (P<0.05). The level of IL-10 in volunteers with active lesion (healing form) with SLA stimulation and without stimulation was 261.3 ± 123.6 and 176.7 ± 142.9, respectively, and the difference was not significant. There was no significant difference between the level of IL-10 in healthy volunteers and active lesion (non-healing form) after stimulation, also there was no significant difference between the level of IL-10 in volunteers with history of CL (healing form) and healthy volunteers after stimulation. There was no significant difference between the level of IL-10 in volunteers with history of CL (healing form) and volunteers with active lesion (non-healing form) after stimulation but there was a significant (*p*<0.05) difference between the level of IL-10 in volunteers with history of CL and volunteers with active lesion after stimulation. There was a significant (*p*<0.005) difference between the level of IL-10 in volunteers with history of CL (non-healing form) and volunteers with active lesion (healing form) after stimulation (Fig. 1B).

### Level of IL-5 in volunteers’ PBMC, stimulated with SLA.

The volunteers’ PBMC were collected and stimulated with SLA and then the supernatants were used to titrate the level of IL-5, the level of IL-5 in healthy volunteers with SLA stimulation and without stimulation with SLA was 256.1 ± 170.2 and 158.7 ± 41.9 and respectively, the difference was not significant. The level of IL-5 in volunteers with active lesion (non-healing form) with SLA stimulation and without stimulation was 297.4 ± 164.8 and 191.0 ± 71.3 and respectively, the different was not significant. The level of IL-5 in volunteers with active lesion (healing form) with SLA stimulation and without stimulation was 218.5 ± 89.0 and 181.5 ± 68.5 and, respectively, the different was not significant. The level of IL-5 in volunteers with history of CL (non-healing form) with SLA stimulation and without stimulation was 167.3 ± 44.8 and 128.6 ± 25.7 respectively, and the different was not significant. The level of IL-5 in volunteers with history of CL healing form with SLA stimulation and without stimulation was 243.0 ± 138.4 and 170.6 ± 101.3, respectively, the different was not significant. There was no significant difference between the levels of IL-5 in different groups after stimulation (Fig. 1C).

## DISCUSSION

Leishmaniasis cure and protection depend upon development of Th1 type of immune response. Usually CL heals spontaneously even without any treatment but in rare cases CL lesion does not heal on expected time and a non-healing lesion (s) develops which is refractory to all types of available modalities. Non-healing form of CL is more often caused by *L. tropica*, but there are reports of non-healing lesion due to *L. major* (11, 12). There are many studies on immune response evaluation in leishmaniasis but yet there is no information available about the surrogate marker(s) of healing and protection in human leishmaniasis (10, 11). An induction of protection in intracellular parasitic infection such as mycobacterial infection, trypanosomiasis, toxoplasmosis and leishmaniasis depends upon development of Th1 type of immune response (19). The immune response development in leishmaniasis is complicated; there are many studies on the immune response evaluation of CL patients (9–12).

The results of the current study showed that the level of IFN-γ in volunteers with history of CL was significantly higher than the volunteers with active lesion which is different to another study (11), which showed that the level of IFN-γ in the culture supernatants of PBMC in CL patients with active lesion was similar to the IFN-γ level in volunteers with history of CL. In the current study, the level of IFN-γ production was significantly higher in patients with active lesion, history of CL than healthy volunteers which is similar to the previous studies (10, 12). In some studies the levels of IFN-γ and CD26 in plasma and PBMC were evaluated as marker of Th1 response and CD30 as a marker of Th2 response; one study using the mentioned markers showed a Th2 response in volunteers with non-healing form of lesion and Th1 response in volunteers with healing form of lesion and in another study there was no significant difference between the level of CD30 and CD26 in plasma of patients with active lesion (non –healing form) and healthy volunteers or volunteers with history of CL (20–23). The level of IFN-γ, before stimulation with SLA was not significantly different between healthy volunteers and other groups of CL active lesion and history of CL, in another word the level of IFN-γ before stimulation with SLA was similar in all groups which is an indication that production of IFN-γ in PBMC culture needs pre-exposure of the volunteer to *Leishmania* antigens; it means that in fact memory cells are responsible for IFN-γ production. The results showed that the level of IFN-γ increased during lesion development and healing process and stays significantly higher than the time of initiation of infection. Although the level of IFN-γ was significantly higher in patients with non-healing form of CL than healthy volunteers but significantly lower than CL patients with healing form of lesion, which might be an indication of development of Th1 type of response in non-healing form of lesion but the response was not as intense as in healing form of CL lesion.

The IL-5 level was higher in PBMC collected from patients with non-healing form of lesion but was not significant, one of the reason is that the sample size was not big enough to show the significant p-value.

IL-10 was originally defined as a Th2 cytokine and a potent inhibitor of Th1 development but nowadays it is clear that IL-10 plays several roles in immune response including induction of protection (24). The level of IL-10 from PBMC of healthy and active lesion (healing/non-healing form) stimulated with SLA was detectable and in most cases higher than no stimulation. IL-10 production in PBMC culture supernatants of volunteers with history of CL stimulated with SLA was significantly higher (P<0.05) than unstimulated. IL-10 is associated with chronicity of the lesion in C57BL/6 mice and in *L. major* infected IL-10 knock out group of C57BL/6 mice, sterile cure occurs with no protection against challenge (25–26), so present of IL-10 is necessary for induction of protection in leishmaniasis. IL-5 levels in supernatants of PBMC collected from healthy volunteers and CL patients with non-healing form of CL was higher than volunteers with history of CL but the difference was not significant which might be due to somehow a mixed Th1/Th2 response. There was no significant production of IL-5 in any group before or after SLA stimulation.

## CONCLUSION

Development of immune response against cutaneous leishmaniasis initiates after exposure to *Leishmania* and the intense of the response increases till cure of the lesion, there is a significant difference between IFN-γ of leishmaniasis patients (all types) and healthy volunteers, also there is a significant difference in IFN-γ of CL patients with healing form and non-healing form.

## References

[1] Alvar J Velez ID Bern C Herrero M Desjeux P Cano J Leishmaniasis worldwide and global estimates of its incidence. PLoS One 2012; 7(5): e35671. 22693548 10.1371/journal.pone.0035671 PMC3365071

[2] World Health Organization (2018). Leishmaniasis. https://www.who.int/leishmaniasis/en/

[3] Vos T Barber RM Bell B Bertozzi-Villa A Biryukov S Bolliger I Global, regional, and national incidence, prevalence, and years lived with disability for 301 acute and chronic diseases and injuries in 188 countries, 1990–2013: a systematic analysis for the Global Burden of Disease Study 2013. Lancet 2015; 386:743–800. 26063472 10.1016/S0140-6736(15)60692-4 PMC4561509

[4] Dowlati Y. Cutaneous leishmaniasis: clinical aspect. Clin Dermatol 1996; 14:425–431. 8889320 10.1016/0738-081x(96)00058-2

[5] Scott P Novais FO. Cutaneous leishmaniasis: immune responses in protection and pathogenesis. Nat Rev Immunol 2016; 16:581–592. 27424773 10.1038/nri.2016.72

[6] Sacks D Noben-Trauth N. The immunology of susceptibility and resistance to *Leishmania major* in mice. Nat Rev Immunol 2002; 2:845–858. 12415308 10.1038/nri933

[7] Choi BS Kropf P. Evaluation of T cell responses in healing and non-healing leishmaniasis reveals differences in T helper cell polarization *ex vivo* and *in vitro*. Parasite Immunol 2009; 31:199–209. 19292771 10.1111/j.1365-3024.2009.01094.x PMC2713858

[8] Kemp K. Cytokine-producing T cell subsets in human leishmaniasis. Arch Immunol Ther Exp (Warsz) 2000; 48:173–176. 10912621

[9] Castellano LR Correia Filho D Argiro L Dessein H Prata A Dessein A Th1/Th2 immune responses are associated with active cutaneous leishmaniasis and clinical cure is associated with strong interferon-γ production. Hum Immunol 2009; 70:383–390. 19480861 10.1016/j.humimm.2009.01.007

[10] Mahmoodi M Khamesipour A Dowlati Y Rafati S Momeni AZ Emamjomeh M. Immune response measured in human volunteers vaccinated with autoclaved *Leishmania major* vaccine mixed with low dose of BCG. Clin Exp Immunol 2003; 134:303–308. 14616791 10.1046/j.1365-2249.2003.02299.x PMC1808866

[11] Ajdary S Alimohammadian MH Eslami MB Kemp K Kharazmi A. Comparison of the immune profile of nonhealing cutaneous leishmaniasis patients with those with active lesions and those who have recovered from infection. Infect Immun 2000; 68:1760–1764. 10722561 10.1128/iai.68.4.1760-1764.2000 PMC97345

[12] Habibi GR Khamesipour A McMaster W Mahboudi F. Cytokine gene expression in healing and non-healing cases of cutaneous leishmaniasis in response to *in vitro* stimulation with recombinant gp63 using semi-quantitative RT–PCR. Scand J Immunol 2001; 54:414–420. 11555409 10.1046/j.1365-3083.2001.00990.x

[13] Sharifi I Poursmaelian S Aflatoonian MR Ardakani RF Mirzaei M Fekri AR Emergence of a new focus of anthroponotic cutaneous leishmaniasis due to *Leishmania tropica* in rural communities of Bam district after the earthquake, Iran. Trop Med Int Health 2011; 16:510–513. 21255206 10.1111/j.1365-3156.2011.02729.x

[14] Bogdan C. Leishmaniasis in rheumatology, haematology and oncology: epidemiological, immunological and clinical aspects and caveats. Ann Rheum Dis 2012;71(Supp II):i60–i66. 22460140 10.1136/annrheumdis-2011-200596

[15] Khamesipour A. Therapeutic vaccines for leishmaniasis. Expert Opin Biol Ther 2014;14:1641–1649. 25077737 10.1517/14712598.2014.945415

[16] Goto H Lindoso JAL. Current diagnosis and treatment of cutaneous and mucocutaneous leishmaniasis. Expert Rev Anti Infect Ther 2010; 8: 419–433. 20377337 10.1586/eri.10.19

[17] Mohammadi AMA Khamesipour A Khatami A Behnia M Eskandari SE. Cutaneous leishmaniasis in suspected patients referred to the center for research and training in skin diseases and leprosy, Tehran, Iran from 2008 to 2011. Iran J Parasitol 2013;8: 430–436. 24454437 PMC3887245

[18] Scott P Pearce E Natovitz P Sher A. Vaccination against cutaneous leishmaniasis in a murine model. II. Induction of protective immunity with a soluble extract of promastigotes. J Immunol 1987; 139:221–227. 3495599

[19] Salgame P. Host innate and Th1 responses and the bacterial factors that control *Mycobacterium tuberculosis* infection. Curr Opin Immunol 2005; 17:374–380. 15963709 10.1016/j.coi.2005.06.006

[20] Jafari-Shakib R Shokrgozar MA Nassiri-Kashani M Malakafzali B Nikbin B Khamesipour A. Plasma sCD26 and sCD30 levels in cutaneous Leishmaniasis. Acta tropica 2009;109: 61–63. 18983807 10.1016/j.actatropica.2008.09.018

[21] Jafari-Shakib R Ajdary S Mohtasham Amiri Z Miramin Mohammadi A Nourijelyani K Mortazavi H CD26 expression on CD4+ T cells in patients with cutaneous Leishmaniasis. Clin Exp Immunol 2008; 153: 31–36. 18460019 10.1111/j.1365-2249.2008.03666.x PMC2432094

[22] Tolouei S Ghaedi K Khamesipour A Akbari M Baghaei M Hasheminia SJ IL-23 and IL-27 levels in macrophages collected from peripheral blood of patients with healing vs non-healing form of cutaneous leishmaniasis. Iran J Parasitol 2012; 7:18–25. 23133467 PMC3488816

[23] Ajdary S Jafari-Shakib R Riazi-Rad F Khamesipour A. Soluble CD26 and CD30 levels in patients with anthroponotic cutaneous leishmaniasis. J Infect 2007; 55:75–78. 17241668 10.1016/j.jinf.2006.12.005

[24] Schwarz T Remer KA Nahrendorf W Masic A Siewe L Müller W T cell-derived IL-10 determines leishmaniasis disease outcome and is suppressed by a dendritic cell based vaccine. PLoS Pathog 2013; 9(6):e1003476. 23825956 10.1371/journal.ppat.1003476 PMC3694851

[25] Anderson CF Lira R Kamhawi S Belkaid Y Wynn TA Sacks D. Il-10 and TGF-β control the establishment of persistent and transmissible infections produced by leishmania tropica in C57BL/6 mice. J Immunol 2008; 180: 4090–4097. 18322219 10.4049/jimmunol.180.6.4090

[26] Belkaid Y Hoffmann KF Mendez S Kamhawi S Udey MC Wynn TA The role of interleukin (IL)-10 in the persistence of *Leishmania major* in the skin after healing and the therapeutic potential of anti-IL-10 receptor antibody for sterile cure. J Exp Med 2001; 194:1497–1506. 11714756 10.1084/jem.194.10.1497 PMC2193677

